# Leprous lesion presents enrichment of opportunistic pathogenic bacteria

**DOI:** 10.1186/s40064-015-0955-1

**Published:** 2015-04-18

**Authors:** Paulo ES Silva, Patrícia S Costa, Marcelo P Ávila, Maria Luíza S Suhadolnik, Mariana P Reis, Ana Paula C Salgado, Mário FR Lima, Edmar Chartone-Souza, Andréa MA Nascimento

**Affiliations:** Departamento de Biologia Geral, Instituto de Ciências Biológicas, Universidade Federal de Minas Gerais, Av. Antônio Carlos 6627, Belo Horizonte, CEP: 31270-901 Minas Gerais Brazil; Laboratório Hermes Pardini, Rua Aimorés, 66 Belo Horizonte, CEP: 30140-070 Minas Gerais Brazil

**Keywords:** Leprosy, 16S rRNA gene, Skin, Diversity, Microbiota

## Abstract

Leprosy is a chronic infectious disease that remains a major challenge to public health in endemic countries. Increasing evidence has highlighted the importance of microbiota for human general health and, as such, the study of skin microbiota is of interest. But while studies are continuously revealing the complexity of human skin microbiota, the microbiota of leprous cutaneous lesions has not yet been characterized. Here we used Sanger and massively parallel small sub-unit rRNA (SSU) rRNA gene sequencing to characterize the microbiota of leprous lesions, and studied how it differs from the bacterial skin composition of healthy individuals previously described in the literature. Taxonomic analysis of leprous lesions revealed main four phyla: Proteobacteria, Firmicutes, Bacteroidetes, and Actinobacteria, with Proteobacteria presenting the highest diversity. There were considerable differences in the distribution of Proteobacteria, Bacteroidetes, Firmicutes, and Actinobacteria, with the first two phyla enriched and the other markedly diminished in the leprous lesions, when compared with healthy skin. *Propionibacterium*, *Corynebacterium* and *Staphylococcus*, resident and abundant in healthy skin, were underrepresented in skin from leprous lesions. Most of the taxa found in skin from leprous lesions are not typical in human skin and potentially pathogenic, with the *Burkholderia*, *Pseudomonas* and *Bacillus* genera being overrepresented. Our data suggest significant shifts of the microbiota with emergence and competitive advantage of potentially pathogenic bacteria over skin resident taxa.

**Electronic supplementary material:**

The online version of this article (doi:10.1186/s40064-015-0955-1) contains supplementary material, which is available to authorized users.

## Introduction

*Mycobacterium leprae* is the causative agent of leprosy, an ancient chronic infectious disease and may have severely debilitating physical, social, and psychological consequences. The skin, the peripheral nerves, the nasal mucosa, eyes, and the reticulum-endothelial system are the preferred target sites for this pathogen. The disease displays a spectrum of clinical manifestations, such as lepromatous (multibacillar) and tuberculoid (paucibacillar) leprosy, which are attributed to the host immune response. It still remains a stigmatizing disease (Nascimento 2013; Degang et al. 2014). This neglected tropical disease has a close relationship with poverty, being a major challenge to public health in countries where it remains endemic. Data reported by the (World Health Organization 2013) revealed that, in 2012, around 122 countries presented cases of leprosy with India showing the highest number of cases (134,752) followed by Brazil (33,303).

Increasing evidence is continuously bringing to light the importance of microbiota for human general health, including its essential role in physiology, and in our immune responses and metabolism (Cho & Blaser 2012). Thus, the human microbiome has been referred to as a forgotten organ (Morgan & Huttenhower 2012) New sequencing technologies are transforming the study of microbial diversity and have revealed that the human skin harbors a complex microbiota. Previous studies highlight that the human skin microbiome is diverse and personalized (Grice et al. 2009; Costello et al. 2009). Indeed, among the 19 bacterial phyla found so far by these studies, special attention goes to the Actinobacteria, Firmicutes, Proteobacteria, and Bacteroidetes phyla, which are consistently reported and account for 99% of the 16S rRNA gene sequences. These studies have also uncovered the genera *Corynebacterium*, *Propionibacterium*, and *Staphylococcus* as abundant resident microbiota of human skin.

Other microbiome studies have provided insights into the delicate balance between skin health and disease (Grice et al. 2009; Costello et al. 2009; Gao et al. 2008; Kong et al. 2012). Studies on the skin microbiota of individuals with non-infectious diseases, such as atopic dermatitis and psoriasis, have revealed a variation in the bacterial composition of the skin of these patients when compared to healthy persons (Gao et al. 2008; Kong et al. 2012; Dekio et al. 2007). In comparison to healthy individuals, atopic dermatitis patients show a higher abundance of *Stenotrophomonas maltophilia*, and a lower abundance of *Propionibacterium acnes* and *Staphylococcus* sp., both are resident skin bacteria (Dekio et al. 2007). In patients with psoriatic lesions, the most abundant phyla were Firmicutes and Actinobacteria, with Firmicutes significantly overrepresented while Actinobacteria is underrepresented comparing to healthy skins (Gao et al. 2008). However, studies on the bacterial community composition of the skin of individuals with leprosy are still missing.

In this study we characterized the skin microbiota of leprous lesions to determine whether it differs from the skin bacterial composition of healthy individuals by Sanger and massively parallel small sub-unit rRNA (SSU) rRNA gene sequencing. The data presented herein have important implications to foster research about the role of skin microbiota in leprosy.

## Materials and methods

### Ethics statement

The study was approved by the Universidade Federal de Minas Gerais Research Ethical Committee with approval number CAAE - 0709.0.203.000-11. The leprous skin samples were obtained from Hermes Pardini pathological anatomy laboratory of Belo Horizonte, Brazil. The samples were rendered anonymized for researchers before its use.

### Specimen and DNA extraction

Samples studied were archival formalin-fixed paraffin embedded sections of lepromatous leprosy lesion skin. The skin biopsies measuring approximately 3 × 3 mm were collected from volar forearm prior to antimycobacterial treatment. Before proceeding to the DNA extraction the paraffin blocks were washed with ethanol 70%, for decontamination, and a new blade was placed in the microtome. The first sections were discarded and the next ones were used for DNA extraction. DNA extraction was carried out according to a procedure modified from Coura et al. (2005). After the procedure of digestion with proteinase K, DNA extraction was continued using phenol-chloroform as described by Sambrook et al. (1989). Total DNA was quantified by absorbance at 260 nm using a NanoDrop Spectrophotometer (NanoDrop Technologies). DNA purity was assessed using the A260/A280 ratio. The DNA was stored at −20°C until further processing. We also included in the analysis the results from samples previously obtained from psoriasis and atopic dermatitis patients and from healthy persons (Grice et al. 2009; Costello et al. 2009; Gao et al. 2008; Kong et al. 2012; Dekio et al. 2007).

### PCR amplification of the 16S rRNA gene, cloning and Sanger sequencing

The bacterial 16S rRNA gene fragment was amplified using touchdown PCR according to Freitas et al. (2008), with the conserved primer set 8f (5′-AGAGTTTGATCMTGGCTCAG-3′) and 907r (5′-TACGGHTACCTTGTTACGACTT3-′) (Lane 1991). The amplicons were gel-purified using the QIAquick Gel extraction kit (Qiagen, Hilden, Germany), cloned into the vector pJET1.2/blunt (Fermentas, Canada) according to the manufacturer’s instructions, and transformed into electrocompetent *Escherichia coli* DH5α. The 16S rDNA fragments were sequenced bidirectionally using the pJET1.2 forward and reverse primers and an ABI Prism 3130 DNA sequencer (Applied Biosystems, Foster City, CA).

It is worth noticing that of the initial 20 samples, only six were amplified successfully. Of these, only one sample produced a satisfactory final PCR product yield for the 16S rRNA gene library construction, despite our strenuous attempts (10). Indeed, in addition to the 8f and 907r, we also used primers 27f (5′-AGAGTTTGATCCTGGCT CAG-3′) and 1492r (5′-GGTTACCTTGTTACGACTT-3′) (Lane et al. 1985) and diverse Taq polymerase, to no avail.

### Phylogenetic analysis

Sequences were assembled using Linux programs Phred/Phrap/Consed (http://www.phrap.org/phredphrapconsed.html). Chimeric sequences were identified using Bellerophon (Huber et al. 2004). Good’s coverage (Good 1953) and rarefaction curves were calculated for operational taxonomic units (OTUs) with an evolutionary distance of 0.03, using the DOTUR program (Schloss & Handelsman 2005). The OTUs were compared with available databases using the BLASTn search tool from GenBank (http://www.ncbi.nlm.nih.gov/). Sequence alignment and phylogenetic relationships were inferred with ARB (Ludwig et al. 2004; Pruesse et al. 2007) using the neighbor-joining algorithm (http://www.arb-home.de). The bootstrap consensus tree inferred from 500 replicates (Felsenstein 1985) was taken to represent the evolutionary history of the taxa analyzed. The nucleotide sequences generated were deposited into the GenBank database under the accession numbers KJ 022641 to KJ 022699.

### V3-V4 hypervariable regions PCR amplification and massively parallel sequencing

Amplification of the V3-V4 hypervariable regions was performed using the region-specific bacterial primers S-D-Bact-0341-b-S-17 forward 5′-CCTACGGGNGGCWGCAG-3′ and S-D-Bact-0785-a-A-21 reverse 5′-GACTACHVGGGTATCTAATCC-3′ (Kozich et al. 2013), with Illumina adapters added. Barcoded amplicons were generated using KAPA HiFi HotStart ReadyMix (KAPA, Woburn, MA, USA) and purified using AMPure XP beads (Agencourt Bioscience, Beverley, MA, USA). Sequencing was performed using the MiSeq platform (Illumina, Inc., San Diego, CA, USA) according to manufacturer’s instructions.

### Bioinformatics analysis

16S rRNA microbiota primary data analysis was performed with PRINSEQ (stand alone lite version, http://prinseq.sourceforge.net/) where quality-based trimming was done. Reads with N’s or an overall mean Q-score < 25 were discarded. The resulting fasta file was also screened for ambiguous base and homopolymers by MOTHUR v.1.33.0 (http://www.mothur.org). Chimeras were detected using the UCHIME algorithm (http://drive5.com/uchime). Moreover, OTUs and taxonomic classification were determined using the closed-reference strategy implemented in QIIME 1.8 (Caporaso et al. 2010), with reads clustered at 97% of similarity, against the Greengenes reference database (release August 2013). The nucleotide sequences were submitted to Sequence Read Archive (SRA) with the accession number of PRJNA267733.

## Results

**Figure 1:**
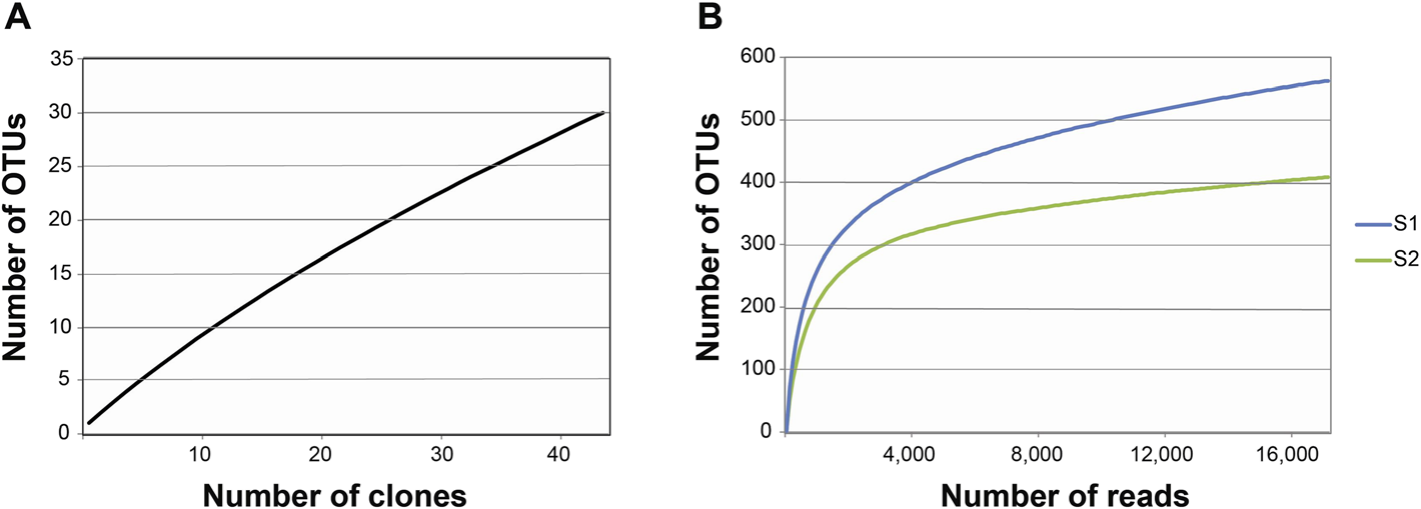
Rarefaction curves on the dataset of the samples from leprous skin lesion. **A**. Sanger sequencing and **B** massively parallel sequencing.

The bacterial composition of leprous lesions was investigated using traditional and massively parallel sequencing. We first studied the bacterial community by constructing and Sanger sequencing a 16S rRNA gene library of one skin biopsy sample. Rarefaction analysis indicated that diversity was reasonably well sampled, as evidenced by the non-asymptotic curve presented in Figure 1, a result concordant with the Good’s coverage data (73%). A total of 88 clones were randomly picked and sequenced. Fifty-nine 16S rRNA gene sequences were obtained after quality control and removal of chimeric sequences. For phylogenetic analysis, only partial sequences of ~600 nucleotide long of the 16S rRNA genes were used, which spanned the V2 to V4 hypervariable regions corresponding to *Escherichia coli* K12.

**Figure 2:**
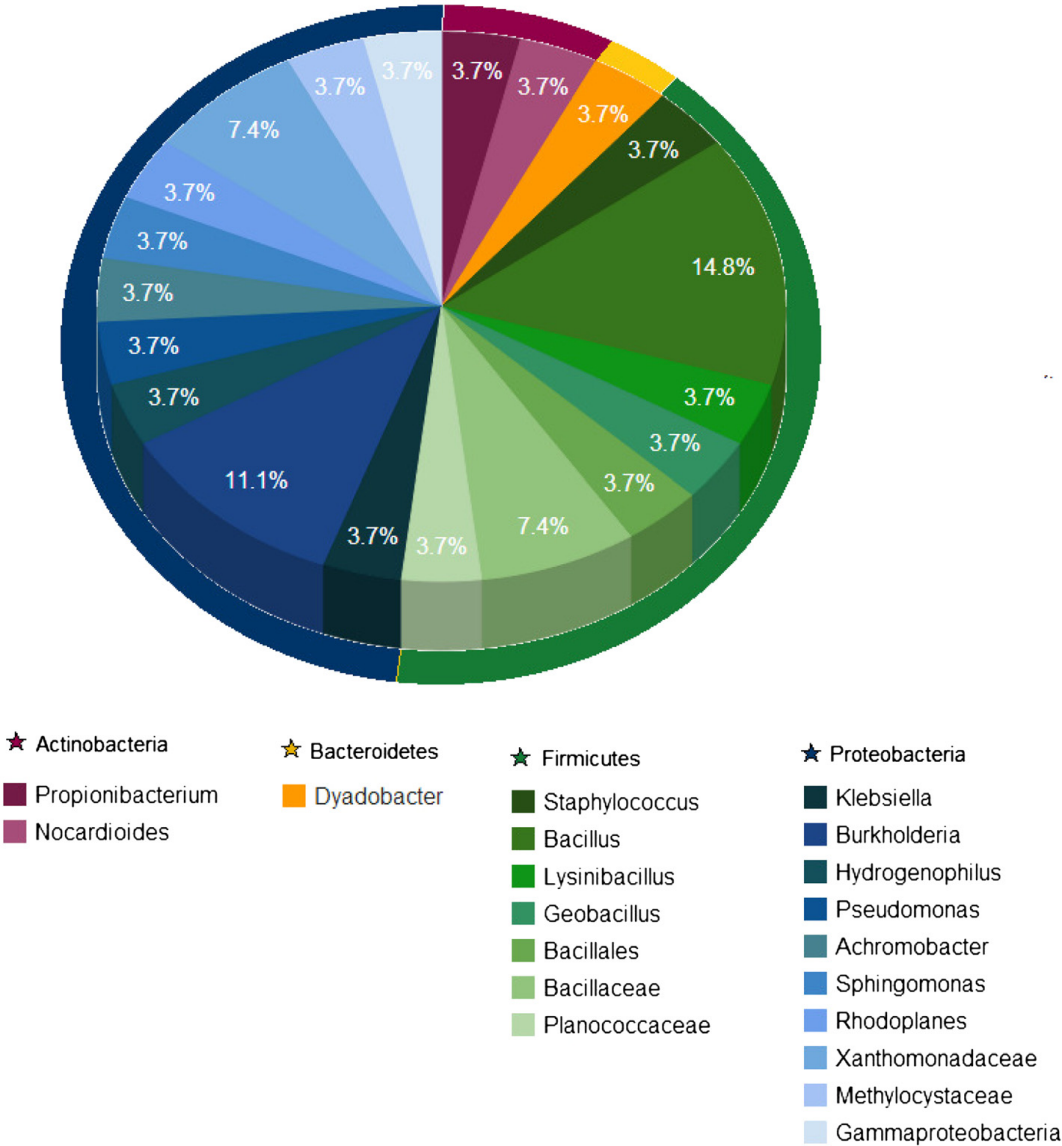
Relative abundance of taxa observed in bacterial 16S rRNA gene library from leprous skin lesion, based on Sanger sequencing.

**Figure 3:**
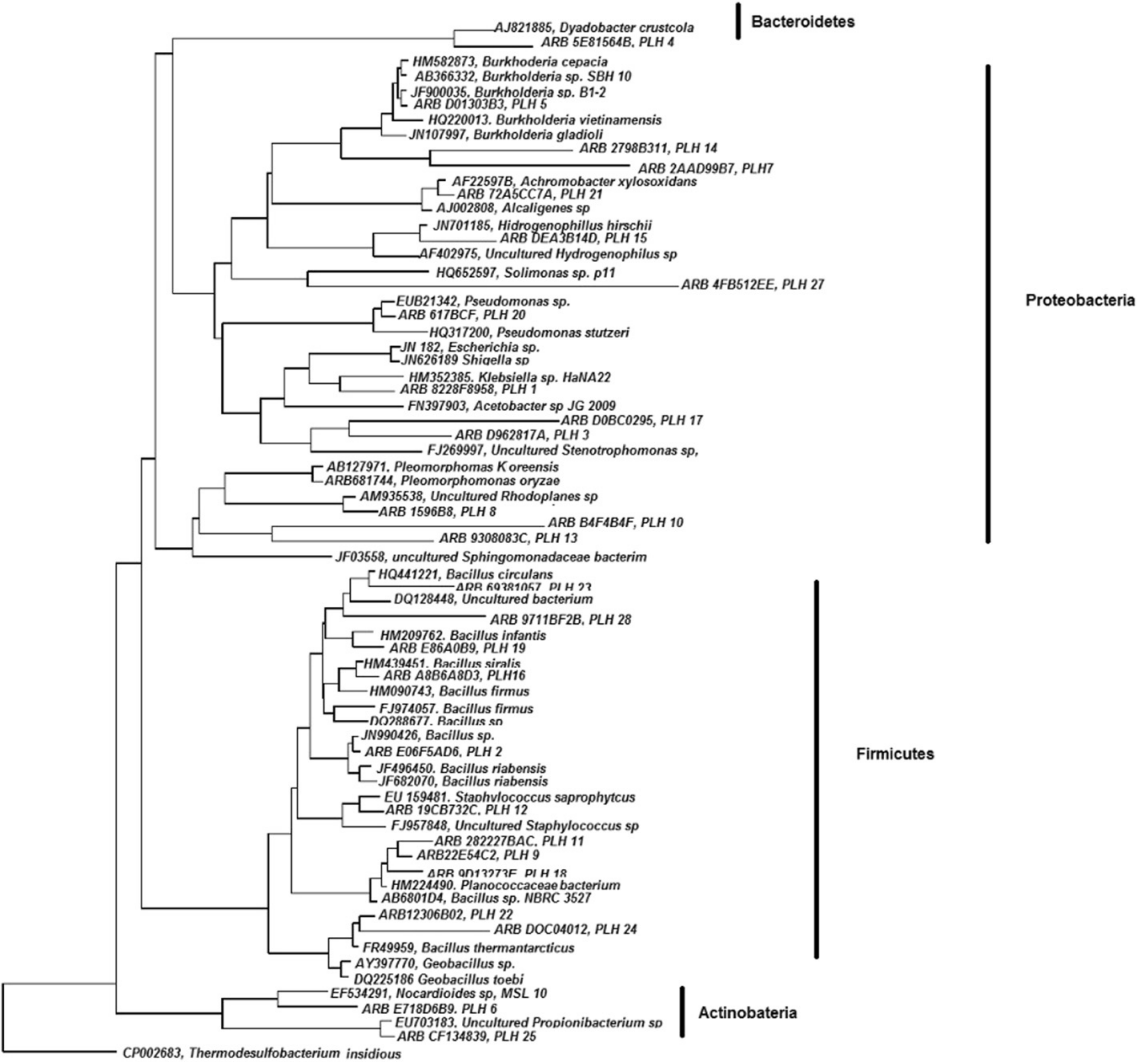
Phylogenetic tree, constructed using the neighbor-joining method, shows the affiliation of bacterial OTUs from leprous skin lesions.

To determine the bacterial diversity associated with leprosy, the 16S rDNA clone sequences were analyzed phylogenetically. They were distributed into 27 OTUs spanning four bacterial phyla. The relative abundance of the phylogenetic groups as well as the resulting phylogenetic tree are shown in Figures 2 and 3, respectively.

The largest fractions of the clone library were represented by Proteobacteria (48%) and Firmicutes (41%). Actinobacteria, the most prevalent and diverse phylum in normal skin from healthy persons, was underrepresented in the leprous sample analyzed. Bacteroidetes phylum comprised the smallest fraction (Figure 2).

Proteobacteria was characterized by a broad diversity with the most abundant OTU classified at genus level as *Burkholderia*, and the other OTUs as *Klebsiella*, *Hydrogenophilus*, *Pseudomonas*, *Achromobacter*, *Sphingomonas*, and *Rhodoplanes* were evenly abundant (3.7% each). In contrast, Bacteroidetes was represented by a single genus, *Dyadobacter*. The most abundant Firmicutes OTU was the *Bacillus* genus (14.8%), whereas *Propionibacterium* and *Staphylococcus*, typical resident bacteria of normal skin, were less abundant (3.7% each) (Figure 2). The order *Actinomycetales*, which harbors the species *Mycobacterium leprae*, was represented in our study by the genus *Nocardioides* (Figure 2).

Most OTUs displayed relationships with sequences of culturable bacteria obtained from a wide range of environments, from volcanic to copper mining. Only two OTUs were related to culturable bacteria from human body sites, including skin and vagina. Furthermore, eight OTUs for which no corresponding cultured genera are known, included sequences most similar to the class Gammaproteobacteria (1 OTU), order Bacillales (1 OTU) and families Bacillaceae (2 OTUs), Planococcaceae (1 OTU), Methylocystaceae (1 OTU), and Xanthomonadaceae (2 OTUs), and thus may represent novel bacterial taxa (Additional file 1: Table S1).

**Figure 4:**
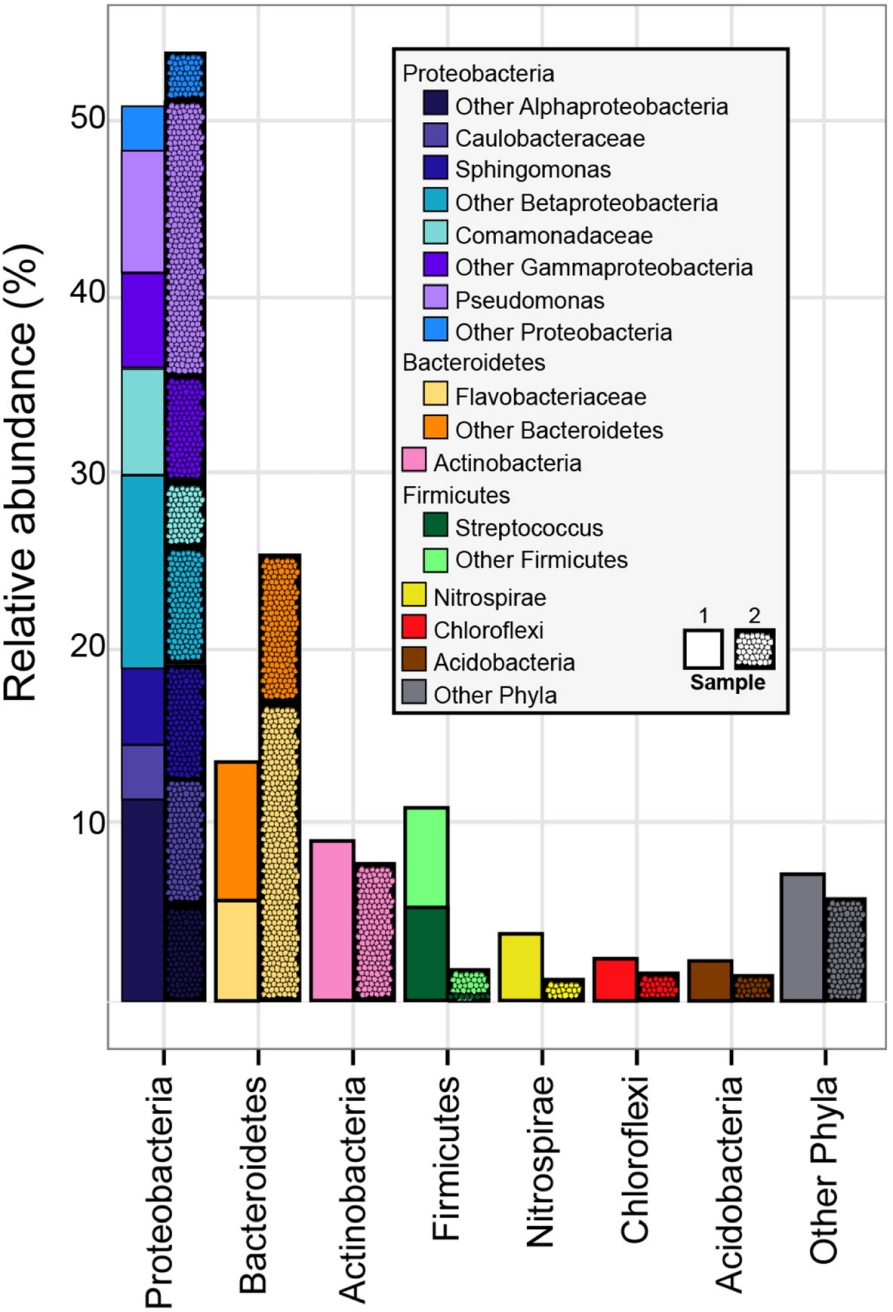
Relative abundance of taxa observed in two leprous lesions samples, based on massively parallel sequencing. V3-V4 tags are grouped into phylum. Each phylum bar is broken down when a particular taxonomic group dominated the phylum. Other phyla are: AC1, Armatimonadetes, Chlorobi, Cyanobacteria, Elusimicrobia, Fusobacteria, Gemmatimonadetes, GN02, GN04, OD1, OP1, OP11, OP3, OP8, Planctomycetes, Spirochaetes, TM7 and WS3.

To reveal the fine details of leprous lesions microbiota we conducted massively parallel sequencing on the V3-V4 hypervariable regions of the 16S rRNA gene (abbreviated henceforth as V3-V4 tag). V3-V4 tag of two skin biopsy samples yielded a total of 80 514 high quality reads (17 038 of S1 and 63 476 of S2), with the average read length of 455 bp. The Good’s coverage values (99.2% and 99.8%) and rarefaction curves (Figure 1) obtained with an evolutionary distance of 0.03 indicated that the diversity was thoroughly uncovered. The reads were clustered into 1 084 OTUs (562 of S1 and 522 of S2), spanning a total of 27 phyla (Figure 4). Proteobacteria, Bacteroidetes, Actinobacteria and Firmicutes represented 88.3% of all reads. The main four phyla were the sole found in the Sanger sequencing. The minor bacterial phyla were Acidobacteria, Chloroflexi and Nitrospirae, accounting for 5.5% of all reads. The group “other bacteria” comprised Gemmatimonadetes, Cyanobacteria, Verrucomicrobia, OP3, GN04 Elusimicrobia, Planctomycetes, Fusobacteria, among others, representing 10.7% of the OTUs.

The most abundant phylum was Proteobacteria, which comprised more than half of all reads. Reads affiliated with Gamma- and Alphaproteobacteria predominated, constituting 72.5% of all proteobacterial reads. The remaining reads belonged to Beta- (22.1%), Delta (5.4%) and Epsilonproteobacteria (0.0001%). As in the Sanger sequencing, Proteobacteria harbored wide diversity of 16S rRNA reads, totalizing 50 families and 79 genera. Among the 10 top proteobacterial taxa there were representatives from different families or genera, namely, *Pseudomonas* (32.4%), *Sphingomonas* (13.7%), Caulobacteraceae (15%), Xanthomonadaceae (5.3%), Alcaligenaceae (2.5%), *Proteus* (1.7%), *Gallionella* (3.9%), Comamonadaceae (9.8%), *Chromobacterium* (1.3%) and *Crenothrix* (2.5%), accounting for 88.1% of all proteobacterial reads.

In contrast to Sanger sequencing, Bacteroidetes was the second most abundant phylum. Seventy-one percent of all Bacteroidetes-associated reads were affiliated with the Flavobacteriaceae family. Other taxa found were *Sphingobacterium*, *Leadbetterella*, and *Elizabethkingia meningoseptica*.

Streptococcaceae, Planococcaceae, Bacillaceae, Ruminococcaceae and Staphylococcaceae were the members dominants of Firmicutes. The genus *Streptococcus* comprised almost half of all Firmicutes-associated reads, whereas representation of the genus *Staphylococcus* was very low (0.2%). Actinobaceria were underrepresented in the samples, in agreement with the Sanger sequencing. Within Actinobacteria, the Micrococaceae and Intrasporangiaceae families were the most abundant and comprised 36.6% and 16.6% of total reads. Nevertheless, *Propionibacterium* (0.7%) and *Corynebacterium* (0.4%) were also found in lower abundance. It should be noted that *Mycobacterium* were represented by a few reads (0.0007%).

## Discussion

Leprosy is a stigmatizing disease because of the deformation caused by the skin lesions displayed by infected individuals. Recent investigations have highlighted the role of skin microbiota at the interface of health and disease (Cho & Blaser 2012). Thus, accurate characterization of skin bacterial communities is an important challenge in the search for possible links between microbiota changes and disease. The current study used Sanger and massivelly parallel SSU rRNA sequencing approaches to characterize the skin microbiota of individuals with leprosy and attempted to determine how it differs from the bacterial skin composition of healthy individuals. The sequencing depth in this study revealed relatively rare members of the skin bacterial community that collectively could have a negative implication on health.

Leprous skin lesion revealed four dominant phyla represented by Proteobacteria, Bacteroidetes, Firmicutes and Actinobacteria. The same phyla were found in skin from psoriasis and atopic dermatitis patients and from healthy persons (Grice et al. 2009; Costello et al. 2009; Gao et al. 2008; Kong et al. 2012; Ludwig et al. 2004). However, the distribution of these phyla in the leprous lesion studied here was distinct from that reported in these studies. Indeed, while Actinobacteria is the most abundant and diverse phylum in healthy skin, with distribution ranging from 27% to 52% (Grice et al. 2009; Costello et al. 2009; Blaser et al. 2013), in leprotic skin it was markedly underrepresented (Figure 4). Actinobacteria was also underrepresented (37.3%) in psoriatic skin patches compared to healthy skin from the same patients (47.8%) and from unaffected controls 47.6%; (Gao et al. 2008). As already suggested by Gao et al. (2008) for psoriasis, the observed reduction in Actinobacteria representation in the leprous lesion may be the result of disordered ecological niches of the diseased skin, turning it inhospitable to these bacteria. Interestingly, in the leprous lesion *Propionibacterium* and *Corynebacterium* were scarcely detected, in contrast to their known dominant presence in normal skin (Grice et al. 2009; Costello et al. 2009). Therefore, it is likely that Actinobacteria and in particular the *Propionibacterium* and *Corynebacterium* genera may have a protective role in normal skin that is diminished in leprous lesions. Underrepresentation of *Propionibacterium* species has also been observed in the psoriatic lesions either in skin swab or biopsy (Gao et al. 2008; Fahlén et al. 2012). The absence (Sanger sequencing) or a fewer (massive parallel sequencing) of *M. leprae*-related sequences found in our study is in agreement with Fiallo et al. (Fiallo et al. 1992), who reported that the formalin fixation of tissues can affect the PCR for *M. leprae* DNA, leading to a significant reduction in the detection of *M. leprae*. Although the Firmicutes phylum was less abundant, *Streptococcus* was enriched in leprous lesion. According to Dekio et al. (2007), they are considered to reside only in infected lesions human skin. Another interesting finding was the low abundance of *Staphylococcus* species, which densely colonize the skin, and has been considered a commensal in healthy skin (Iwase et al. 2010).

Proteobacteria and Bacteroidetes, the two other major phyla inhabiting skin of healthy persons, were significantly overrepresented in the leprous lesion (Figure 4). Indeed, in healthy persons the distribution of Proteobacteria ranges from 10 to 33% and that of Bacteroidetes ranges from 2.4 to 10% (Grice et al. 2009; Costello et al. 2009; Gao et al. 2008; Blaser et al. 2013).

Our data revealed that the *Burkholderia* (Sanger sequencing) and *Pseudomonas* (V3-V4 tag) genera were enriched and the most abundant. We also found the minor genera *Nocardioides*, *Lysinibacillus*, *Geobacillus*, *Rhodoplanes*, *Gallionella*, *Phycicoccus*, and *Dyadobacter*; to our knowledge the first identification of such members in human skin. It is possible that leprous lesions impair the skin barrier protection and facilitate the access of bacteria normally absent in healthy skin.

## Conclusion

Here we describe for the first time the taxonomic diversity of the microbiota of the leprous lesion. Sanger and massively parallel sequencing of leprous lesions provided the same phylum-level representation of human skin, that account for 99% of the 16S rRNA gene sequences (Actinobacteria, Proteobacteria, Firmicutes and Bacteroidetes). However, rare and different taxa arise due to a massive increase in the sequencing depth. Our results extend the findings of others by demonstrating that leprous lesion harbors a phylum-level diversity much more thus far known from the healthy skin microbiota. Significant shifts of the microbiota seem to favor the colonization of potentially pathogenic bacteria, negatively impacting the abundance of bacteria that populate healthy skin. The comprehensive current knowledge on complexity in the composition of the microbiota is raising speculation on its correlation with the evolution of this disease. Thus, instead of a single organism being the sole causative agent of a given pathology, as proposed by Koch, disease may be a result of complex interactions among the bacterial community and between the microbiota and its local environment. With this speculation in mind, the current study can be used as a baseline for further research aiming to determine the contribution of bacteria other than *M. leprae* in triggering leprosy.

## Additional file

Additional file 1: Table S1. Phylogenetic affiliation and distribution of bacterial clones analyzed from leprous skin lesion.
