# Supplementary material for: Leprous lesion presents enrichment of opportunistic pathogenic bacteria
Source: Springerplus. 2015 Apr 18;4:187. doi: 10.1186/s40064-015-0955-1 (PMC4405507; doi:10.1186/s40064-015-0955-1)
Supplement: Additional file 1: Table S1. — Phylogenetic affiliation and distribution of bacterial clones analyzed from leprous skin lesion. [file 40064_2015_955_MOESM1_ESM.docx]

Table S1: Phylogenetic affiliation and distribution of bacterial clones analyzed from leprous skin lesion.

| **Phylum** | **OTU** | **Number of clones** | | **Closest sequence/microorganism** | **Number of access** | **Identity** | **Source of closest relative** |
| --- | --- | --- | --- | --- | --- | --- | --- |
| **Actinobateria** | 1 | 2 | *Nocardioides sp* | | AB167236.1 | 96% | Chemostat (ethanol) |
|  | 2 | 1 | *Propionibacterium acnes* | | NR_074675.1 | 99% | Human skin (strain KPA171202) |
| **Bacteriodetes** | 3 | 4 | *Dyadobacter fermentans* | | CP001619.1 | 97% | Maize (rod) |
| **Firmicutes** | 4 | 1 | *Staphylococcus epidermidis* | | KC213963.1 | 99% | Intestine of fish (Rutilus rutilus) |
|  | 5 | 1 | *Bacillus siralis* | | HM439461.1 | 99% | Fermented onion |
|  | 6 | 1 | *Planococcaceae bacterium TPD42* | | HM224490.1 | 98% | Frozen river water |
|  | 7 | 1 | *Bacillus sp. CNJ905 PL04* | | DQ448753.1 | 99% | Marine sediment |
|  | 8 | 12 | *Lysinibacillus sp* | | JX217747.1 | 99% | Fermented soybeans |
|  | 9 | 1 | *Geobacillus thermantarcticus* | | FN428692.1 | 99% | Not described |
|  | 10 | 2 | *Bacillus sp. M71_N104b* | | FM992794.1 | 97% | Aquamarine |
|  | 11 | 1 | *Bacillus circulans* | | FJ581445.1 | 91% | Sludge (polluted station) |
|  | 12 | 1 | *Geobacillus sp. BGSC 20A1* | | AY608980.1 | 92% | Not described |
|  | 13 | 1 | *Bacillus longiquaesitum* | | AM747042.1 | 93% | Soil |
|  | 14 | 4 | *Bacillus sp.* | | DQ249996.1 | 96% | Rock Cave |
| **Proteobacteria** | 15 | 1 | *Pleomorphomonas oryzae* | | AB681744.1 | 93% | Rice |
|  | 16 | 2 | *Klebsiella pneumoniae* | | JN897382.1 | 99% | Water treatment plant (waste) |
|  | 17 | 2 | *Pseudoxanthomonas yeongjuensis* | | NBRC 106397 | 92% | Soil of a field of Korean ginseng |
|  | 18 | 1 | *Burkholderia cenocepacia* | | HQ284839.1 | 99% | Plant leaves |
|  | 19 | 1 | *Hydrogenophilus hirschii* | | FR749905.1 | 96% | Volcano (Solfatara) |
|  | 20 | 1 | *Stenotrophomonas sp. 2R13* | | EF178465.1 | 84% | Rice |
|  | 21 | 2 | *Burkholderia pyrrocinia* | | JQ283970.1 | 99% | Soil (rhizosphere) planting ginger |
|  | 22 | 1 | *Pseudomonas stutzeri* | | KC244183.1 | 99% | Copper Mine Tailings |
|  | 23 | 2 | *Burkholderia cepacia* | | AB695353.1 | 97% | Not described |
|  | 24 | 1 | *Achromobacter sp* | | JN836430.1 | 99% | Soybean Field |
|  | 25 | 9 | *Sphingomonas parapaucimobilis* | | AB680768.1 | 99% | Vaginal swab |
|  | 26 | 1 | *Uncultured gamma proteobacterium* | | EU640742.1 | 97% | Lake Michigan |
|  | 27 | 2 | *Uncultured Rhodoplanes sp.* | | AM935538.1 | 98% | contaminated soil |
